# Guidelines for chronic pain in adult spinal cord injury population: Scoping review

**DOI:** 10.4102/sajp.v80i1.1931

**Published:** 2024-05-06

**Authors:** Tammy-Lee Williams, Conran Joseph, Lena Nilsson-Wikmar, Joliana Phillips

**Affiliations:** 1 Department of Physiotherapy, Faculty of Community and Health Sciences, University of the Western Cape, Cape Town, South Africa; 2 Division of Physiotherapy, Faculty of Health and Rehabilitation Services, Stellenbosch University, Stellenbosch, South Africa; 3 Department of Neurobiology, Faculty of Care Sciences and Society, Karolinska Institutet, Stockholm, Sweden; 4 Department of Research Development and Postgraduate Support, Faculty of Research and Innovation, University of the Western Cape, Cape Town, South Africa

**Keywords:** chronic pain, traumatic spinal cord injury, clinical practice guidelines, pharmacological management, nonpharmacological management

## Abstract

**Background:**

Chronic pain among survivors of spinal cord injury (SCI) hurts physical and mental health. Persons with SCI have demonstrated dissatisfaction with the management of their chronic pain.

**Objectives:**

This study aimed to identify existing clinical practice guidelines for chronic pain in the SCI population.

**Method:**

A scoping review was conducted across various databases available at the University of the Western Cape, in addition to guideline clearing houses (BioMedCentral, Cambridge Journals Online, CINAHL, Cochrane Library, Medline [EbscoHost], Medline [Pubmed], Sabinet Reference, SAGE Journals Online, ScienceDirect, SCOPUS, Wiley Online Library, Springerlink, PubMed, Guideline Central, and Agency for Healthcare Research and Quality). The population consisted of adults with SCI, and the interventions that were included were pharmacological and nonpharmacological management of chronic pain. Guidelines that met the inclusion criteria were critically appraised by two reviewers from this study using the AGREE II instrument. Inter-rater reliability was calculated using SPSS 27, and Cohen’s kappa coefficients were established.

**Results:**

Five articles were included in the data extraction, analysis and appraisal. Two guidelines were rated as high quality, according to the AGREE II tool. In addition, most guidelines focused on neuropathic pain (NeuP) and only one guideline included nociceptive pain and NeuP.

**Conclusion:**

One guideline met the objectives of this scoping review.

**Clinical implications:**

Guidelines developed in the future should include a screening tool to identify the specific type of pain and distinguish peripheral NeuP from central NeuP.

## Introduction

Nontraumatic or traumatic mechanisms can cause spinal cord injury (SCI) (Ahuja et al. 2017). Chronic pain among survivors of SCI has an excessively negative impact on quality of life, activities of daily living, general functioning, sleep, exercise and work across the world (Andresen et al. 2016; Fuseini, Aniteye & Alhassan 2019; Widerström-Noga, Felipe-Cuervo & Yezierski 2001). Chronic pain in SCI consists of neuropathic pain (NeuP), nociceptive musculoskeletal pain (NP) and nociceptive visceral pain (Colloca et al. 2017).

Neuropathic pain consists of central and peripheral NeuP. Central NeuP results from destruction to the central somatosensory nervous system and is identified by the International Spinal Cord Injury Pain (ISCIP) classification as pain present ‘more than three levels below the neurological level of injury’ (Bryce et al. 2012; Finnerup 2013). In addition, central NeuP can be distinguished from peripheral NeuP with changes in sensitivity to prickling sensation and heat (Watson & Sandroni 2016).

Peripheral NeuP results from the destruction of the peripheral somatosensory nervous system. Different mechanisms are proposed for central and peripheral NeuP (Meacham et al. 2017). Furthermore, a combination of central and peripheral NeuP may also be present, which is identified as pain present within the first three levels of the neurological level of injury, with associated damage to the nerve root (Bryce et al. 2012; Finnerup 2013; Hagen & Rekand 2015; Siddall, Taylor & Cousins 1997). Spinal cord injury is often associated with central NeuP. However, persons with SCI of a traumatic nature may experience peripheral NeuP as well (Hatch et al. 2018).

In addition to central and peripheral NeuP, peripheral and central sensitisation may also be present in persons with SCI. Peripheral sensitisation occurs in peripheral NeuP, and central sensitisation occurs in central NeuP.

Peripheral sensitisation is defined by an increase in response to peripheral stimulation due to a lower-than-usual threshold (Wei et al. 2019). If the peripheral sensitisation continues for a prolonged period, central sensitisation results, which causes amplification of pain because of central nervous system mechanisms. It is also known that central sensitisation can continue without peripheral input, especially in chronic pain (Harte, Harris & Clauw 2018; Jensen & Finnerup 2009). This indicates that the management of the initial peripheral sensitisation will no longer be effective when treating central sensitisation.

Qualitative studies indicate that the efficacy of pharmacotherapy for pain relief in the SCI population is limited (Henwood & Ellis 2004; Löfgren & Norrbrink 2012; Widerström-Noga & Turk 2003; Williams et al. 2022). Furthermore, dissatisfaction with current pain management strategies has been expressed among persons with SCI. Persons with SCI have expressed their disinterest in continuing medication for a prolonged period due to their side effects (Norrbrink & Löfgren 2016). In a study by Heutink et al. (2011), persons with SCI indicated that nonpharmacological therapies, such as acupuncture, physiotherapy and exercise, were more effective than pharmacotherapy in relieving chronic pain.

Chronic pain not only has physical ramifications; negative psychological impacts are evident as well. General anxiety, anxiety about future pain relief, lower levels of feeling self-adequate and depression are documented in traumatic spinal cord injury (TSCI) survivors (Andresen et al. 2016; Chin-Ching et al. 2018; Fuseini et al. 2019; Hatefi et al. 2019). Recent studies have indicated that psychological health is also affected by pain. Pain was associated with more anxiety and depression compared to persons without pain in the TSCI population (Al-Owesie, Moussa & Robert 2012). In addition, persons experiencing an increased severity of NeuP also experienced severe depression in the TSCI population (Ghajarzadeh & Saberi 2018).

This scoping review was conducted to map current clinical practice guidelines for chronic pain in the SCI population in light of poor pain relief expressed in qualitative studies, as well as the burden of adverse mental health associated with pain. During the screening process, two reviewers followed the process outlined in Appendix 1, in addition to the date restriction, which guided the retrieval of full texts. Separate from the aim of the scoping review, this study set out to critically appraise clinical practice guidelines.

## Research methods and design

The preferred reporting items for systematic reviews and meta-analysis (PRISMA) extension for scoping reviews were used as a guide for this scoping review. Arksey and O’Malley (2005) proposed the following five phases for scoping and systematic reviews: (1) research question identification, (2) detecting relevant studies, (3) selection of studies based on the inclusion criteria, (4) data extraction and (5) organisation, summarisation and reporting of the results. These phases were followed in this scoping review.

### Phase 1: Research question identification

The review question was framed to ensure that it reflected the population (persons with SCI), concept (guidelines consisting of tools to differentiate chronic pain, that is nociceptive, central and peripheral NeuP as well as guidelines which included pharmacological and nonpharmacological recommendations) and context (clinical practice) of the review (Peters et al. 2015). The following question guided this scoping review: What are the clinical practice guidelines for chronic pain in the SCI population? The objectives of the review were; (1) to determine if guidelines were explicitly aimed at the SCI population, (2) to determine if screening tools were used to classify the type of pain in the guideline, (3) to determine the pharmacological versus nonpharmacological management principles for NeuP, (4) to determine the pharmacological versus nonpharmacological management principles for nociceptive pain and (5) to critically appraise the clinical practice guidelines identified in objectives 1 and 3, using the Appraisal of Guidelines, Research and Evaluation tool, version 2 (AGREE II). The AGREE II instrument assesses the methodological rigour and transparency of guidelines. The AGREE II also provides information as to how guidelines should be reported. It consists of the following six domains, as defined by the AGREE II: ‘Scope and purpose; Stakeholder involvement; Rigour of development; Clarity of presentation; Applicability; and Editorial independence’. The AGREE II tool is valid and reliable, with adequate inter-rater reliability (Brouwers et al. 2010).

### Phase 2: Detecting relevant studies

#### Eligibility criteria

The inclusion criteria for the articles retrieved included publications between 2010 and 2022, articles in English only, and articles documenting chronic pain management in adults (older than 18 years) in SCI survivors only. The restricted date ensures that the most recent and relevant treatments are included in the clinical practice guidelines for chronic pain in the SCI population. Only articles documenting clinical practice guidelines were included. Guidelines documenting acute or subacute pain were excluded.

#### Data sources and search strategy

**FIGURE 1:**
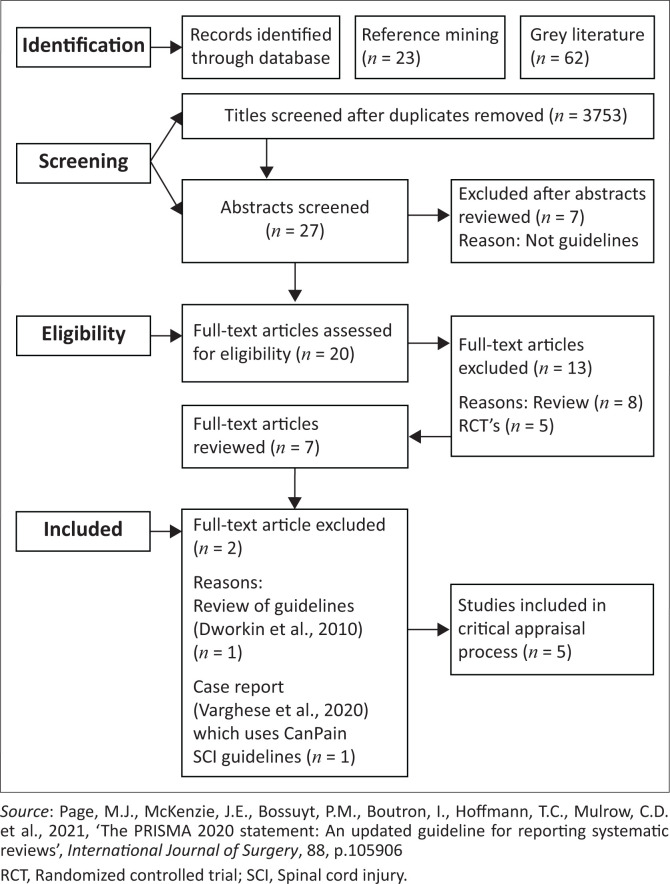
Preferred reporting items for systematic reviews and meta-analysis flowchart.

The search occurred across the University of the Western Cape’s databases: BioMedCentral, Cambridge Journals Online, CINAHL, Cochrane Library, Medline (EbscoHost), Medline (Pubmed), Sabinet Reference, SAGE Journals Online, ScienceDirect, SCOPUS, Wiley Online Library, Springerlink, PubMed, Guideline Central, and Agency for Healthcare Research and Quality. The following MeSH headings and keywords were used: *chronic pain* AND, *clinical practice guidelines*, AND *spinal cord injury.* Certain limits were chosen for each database, and the specifics are mentioned in Appendix 2. A review of the included articles’ reference lists occurred to identify additional articles. A ‘snowball’ technique was used, in which citations within the included articles were searched if they seemed relevant to the scoping review research question (Arksey & O’Malley 2005). As seen in Figure 1, 23 references were identified through reference mining. In addition, other sources such as organisations, conferences and existing networks (grey literature) were also searched. As seen in Figure 1, 62 references were identified among grey literature.

#### Citation management

All citations were imported into the web-based and desktop version of Mendeley Reference Manager (Mendeley Ltd., Elsevier). Additional duplications not automatically removed by Mendeley Reference Manager (Mendeley Ltd., Elsevier) were removed manually. Titles and abstracts were imported into the systematic reviews’ web application (Rayyan QCRI ™) for blind screening by two reviewers from the study.

### Phase 3: Selection of studies

The full texts of the articles that met the criteria were downloaded and reviewed independently by two reviewers from the study. Overall, agreement was present between the two reviewers.

### Phase 4: Data extraction

The data extracted included study characteristics, such as the article’s origin, year of publication, study population and type of pain, as the pharmacological and nonpharmacological guidelines for chronic pain management can be found in Table 1. Data were extracted by one author and reviewed by a second author. This process was followed by discussion. Overall, agreement was present between the two reviewers.

**TABLE 1:** Guidelines extracted from articles.

Authors and year of publication	Origin	Study population	Type of pain	Pharmacological guidelines	Nonpharmacological guidelines
Attal et al. (2010)	France	Central post-stroke pain Multiple sclerosis Spinal cord injury	Chronic central NeuP	First line: **Pregabalin:** 150 mg – 600 mg once a day. **Amitriptyline:** 25 mg – 150 mg once a day. **Gabapentin:** 1200 mg – 3600 mg once a day. Second line: **Tramadol:** 200 mg – 400 mg once a day. **Strong opioids:** (*no dose given*). **Lamotrigine:** (*no dose given*).	
Chetty et al. (2012)	South Africa	SCI Stroke Multiple sclerosis Syringomyelia Spinal infarction Complex regional pain syndrome	Chronic central and peripheral NeuP	First line: **Pregabalin:** 25 mg at night. ■ If required, increase by 25 mg every 2–3 days. ■ Every 3–7 days, increase by 75 mg/day. ■ *Maximum:* two doses of 150 mg – 225 mg, once a day. **Gabapentin:** At night, 100 mg – 300 mg. Alternatively, 100 mg – 300 mg thrice daily. ■ If required, every 1–7 days, increase by 100 mg – 300 mg three times a day. ■ *Maximum*: 1200 mg three times a day. **Amitriptyline:** 10 m – 25 m, at night. ■ If required, increase weekly by 10 mg – 25 mg taken once a day. ■ *Maximum:* 50 mg – 150 mg taken once a day. **Se rotonin and norepinephrine reupatke inhibitors (SNRIs) (duloxetine or venlafaxine)** **Duloxetine**: 30 mg taken once a day. ■ If required, following a week’s waiting period, increase to 60 mg taken once a day. ■ *Maximum*: 60 mg taken twice a day. **Venlafaxine**: 37.5 mg taken once or twice a day. ■ If required, after a week’s waiting period, increase by 75 mg. ■ *Maximum*: 225 mg/day. Second line: Increasing the dose of first-line therapy drugs. Combine Pregabalin with an SNRI or with amitriptyline. Third line: **Tramadol:** 50 mg taken once or twice a day. ■ If required, increase every 3–7 days by 50 mg – 100 mg in divided doses, taken once daily. ■ *Maximum:* 100 mg taken four times a day (400 mg/day). ■ *Maximum:* Persons older than 75 years should not exceed 300 mg daily. **Stronger opioids:** ■ Morphine: 10 mg – 15 mg taken every 4 h, as needed. ■ *If required,* ‘after 1–2 weeks, convert total daily dosage to long-acting opioid analgesic and continue short-acting medication as needed’ (Chetty et al. 2012). ■ *Maximum*: ‘No maximum dosage with careful titration; consider evaluation by pain specialist at relatively high dosages (e.g. morphine at 120 mg q.d. – 180 mg q.d., or equianalgesic dosages of other opioids)’ (Chetty et al. 2012). **First-line therapy combined with opioids**	Spinal cord stimulation Cognitive behavioural therapy TENS Physiotherapy Patient education
Guy et al. (2016) CanPain Guideline	Canada	Spinal cord injury	NeuP	First line: **Pregabalin:** 150 mg – 600 mg taken once a day. **Gabapentin:** 1800 mg – 3600 mg taken once a day. **Amitriptyline:** 10 mg – 25 mg taken once a day. ■ If required, increase to 50 mg per day. Second line: **Tramadol:** 50 mg taken once or twice a day. ■ *Maximum:* 400 mg per day. **Lamotrigine:** Increased to a maximum dose of 400 mg per day. Fourth line: **Oxycodone:** (*no dose given*).	Third line: Transcranial electrical nerve stimulation. Combined visual illusion and transcranial electrical nerve stimulation. Fourth line: Transcutaneous electrical nerve stimulation. Dorsal root entry procedure.
Franz et al. (2019)	Germany	Spinal cord injury	NeuP	First line: **Pregabalin:** 600 mg (two doses, alternatively three doses). ■ If required, increase by 150 mg per day, weekly. **Gabapentin:** ■ Day 1: 100 mg three times a day. ■ Day 2: 200 mg three times a day. ■ Day 3: 300 mg three times a day. ■ If required, increase every other day by 300 mg per day. Second line: **Duloxetine**: 60 mg or 120 mg taken once a day. **Amitriptyline:** 150 mg taken once a day. Third line: **Tramadol:** 50 mg once a day. ■ If required, increase up to a maximum of 100 mg taken once a day. **Oxycodone**: Prescribed with an anticonvulsant (*no dose given*). **Lamotrigine:** Prescribe only for persons with incomplete SCI. ■ 25 mg taken once a day for the first 2 weeks. ■ 50 mg taken once a day over the next 2 weeks. ■ 100 mg once a day or 50 mg taken twice a day for the second month. ■ If required, increase per week by 100 mg. Fourth line: **Venlafaxine:** One single dose of 300 mg. ■ Start with 37.5 mg taken once a day. ■ Increase gradually to 75 mg, taken once daily for the first week. ■ Increase gradually to 150 mg, taken once daily for the second week. ■ Increase gradually to a maximum of 225 mg, taken once a day for the second to sixth week. ■ Increase gradually to a maximum of 300 mg taken once a day for the eighth to tenth week.	Second line: Physical therapy Exercise Physiotherapeutic techniques Psychotherapeutic Third line: tDCs Fourth line: TENS Massage/heat therapy
Franz et al. (2019)	Germany	Spinal cord injury	Chronic nociceptive pain	**Venlafaxine:** One single dose of 300 mg. ■ Start with 37.5 mg taken once a day. ■ Increase gradually to a maximum of 75 mg, once daily, for the first week. ■ Increase gradually to a maximum of 150 mg, taken once a day for the second week. ■ Increase gradually to a maximum of 225 mg, taken once a day for the second to sixth week. ■ Increase gradually to a maximum of 300 mg taken once a day for the eighth to tenth week. **Tramadol:** ■ 50 mg taken once a day. ■ If required, gradually increase to a maximum of 100 mg taken once a day. **Oxycodone:** (*no dose given*). **Botulinum toxin**: intramuscularly (*no dose given*). **Baclofen:** oral application (*no dose given*).	Physical therapy Exercise Physiotherapeutic techniques Psychotherapy TENS (consider on a case-to-case basis if alternative therapy is not available) Massage/heat therapy Hydrotherapy
Schlereth (2020)	Germany	Central neuropathic pain caused by: ischaemia (e.g. insula, thalamus, brain stem), haemorrhage, vascular malformations multiple sclerosis, abscess, myelitis trauma malignancies syringomyelia	Chronic central and peripheral NeuP	First line: **Gabapentin** (peripheral NeuP): three divided doses of 1200 mg – 3600 mg taken once a day. **Pregabalin** (peripheral and central NeuP): two divided doses of 300 mg – 600 mg taken once a day. **Tricyclic antidepressants**: starting at 10 mg – 25 mg at night or in addition to the morning. ■ Increase gradually every 3–5 days by 10 mg – 25 mg. ■ The lower dose is used in elderly persons. ■ *Target dose:* 25 mg – 75 mg taken daily in one dose or divided into bidaily or tridaily doses. **Duloxetine:** 30 mg in the morning. ■ If required, gradually increase it to 60 mg, once daily, after 7–14 days. ■ *Maximum dose:* 120 mg in the morning. Second line: **Lidocaine patch (700 mg):** 1–3 patches applied to the painful region for 12 h. In between applications, there should be a 12-h waiting period. ■ *Maximum dose:* Three patches every 24 h. **Capsaicin (8%):** 60 min on the painful region. ■ *Maximum dose:* simultaneous application of four patches. ■ If required, it can be repeated every 90 days. Third line: **Low-potency opioids such as tramadol and high-potency opioids**: ■ *Maximum dose* for morphine and other opioids: 180 mg taken once a day. ■ *Maximum dose* for oxycodone: 10 mg – 120 mg taken once a day. **Botulinum toxin:** 50–200 units. **Oxcarbazepine:** 300 mg taken once a day. ■ If required, gradually increase to a maximum of 1800 mg taken once daily, in two single doses.	Transcutaneous electrical nerve stimulation (TENS) Psychotherapeutic treatment Multimodal pain therapy Physiotherapy (PT), occupational therapy (OT) and cognitive-behavioral therapy (CBT)

SCI, spinal cord injury; NeuP, neuropathic pain; tDCs, transcranial direct current stimulation.

### Phase 5: Organisation, summarisation and reporting of the results

The various phases of the scoping review are illustrated in a PRISMA flow diagram (Figure 1). Each guideline was independently rated using the AGREE II tool by two appraisers in the study, and a scaled score was determined for each domain, as per the AGREE II methodology (Table 2). SPSS 27 was used to determine inter-rater reliability, using Cohen’s kappa coefficient (Table 2), which is defined as no agreement ≤ 0, none to slight agreement 0.01–0.20, fair agreement 0.21–0.40, moderate agreement 0.41–0.60, substantial agreement 0.61–0.80 and almost perfect agreement 0.81–1.00 (McHugh 2012). Domain scores were categorised according to a previous study assessing guidelines for pain management in patients with low back pain (Doniselli et al. 2018): good (≥ 80%), acceptable (60% – 79%), low (40% – 59%) or very low (< 40%). In the same article, the overall quality of each guideline was scored as follows: when five or more domains were rated > 60%, this indicated a high-quality article; when 3 or 4 domains were rated > 60%, this meant an average quality, and lastly, when less than or only two domains were rated > 60%, this was indicative of a low-quality article. This methodology was also applied in this review.

**TABLE 2:** Using the AGREE II to assess clinical practice guidelines for chronic pain management in patients with spinal cord injury.

Domain	Kappa	Level of agreement	*Z*	Significance	Scaled domain score (%)	Domain quality	The overall quality of the guideline
Guideline 1: Attal et al. (2010)							
Domain 1	0.385	Fair	1.109	0.268	81	Good	Low
Domain 2	0.286	Fair	0.961	0.337	44	Low	
Domain 3	0.231	Fair	1.381	0.167	45	Low	
Domain 4	0.077	None	0.222	0.824	56	Low	
Domain 5	0.091	None	0.286	0.775	25	Very low	
Domain 6	0.600	Moderate	1.177	0.239	21	Very low	
Guideline 2: Chetty et al. (2012)							
Domain 1	0.636	Substantial	1.481	0.139	89	Good	High
Domain 2	0.538	Moderate	1.552	0.121	69	Acceptable	
Domain 3	0.120	Slight	0.671	0.502	68	Acceptable	
Domain 4	1.000	Almost perfect	1.732	0.083	92	Good	
Domain 5	0.391	Fair	1.296	0.195	67	Acceptable	
Domain 6	0.600	Moderate	1.177	0.239	58	Low	
Guideline 3: Franz et al. (2019)							
Domain 1	0.200	Slight	0.346	0.729	97	Good	High
Domain 2	0.000	None	0.000	1.000	75	Acceptable	
Domain 3	0.231	Fair	1.140	0.254	69	Acceptable	
Domain 4	0.636	Substantial	1.481	0.139	89	Good	
Domain 5	0.524	Moderate	1.654	0.098	69	Acceptable	
Domain 6	1.000	Almost perfect	1.000	ns	100	Good	
Guideline 4: Guy et al. (2016)							
Domain 1	1.000	Almost perfect	1.732	0.083	92	Good	Average
Domain 2	0.077	None	0.222	0.824	39	Very low	
Domain 3	0.098	None	0.585	0.559	64	Acceptable	
Domain 4	0.200	Slight	0.346	0.729	97	Good	
Domain 5	0.280	Fair	1.077	0.282	52	Low	
Domain 6	0.333	Fair	0.816	0.414	58	Low	
Guideline 5: Schlereth (2020)							
Domain 1	0.500	Moderate	1.414	0.157	47	Low	Low
Domain 2	0.636	Substantial	1.481	0.139	11	Very low	
Domain 3	0.243	Fair	1.429	0.153	34	Very low	
Domain 4	0.636	Substantial	1.481	0.139	78	Acceptable	
Domain 5	0.600	Moderate	1.664	0.096	21	Very low	
Domain 6	0.600	Moderate	1.177	0.239	71	Acceptable	

### Ethical considerations

This study formed part of a larger research project aimed at creating management principles for chronic pain in the TSCI population. Ethical approval was approved by the Biomedical Research Ethics Committee of the University of the Western Cape (BM20/8/22, 08 December 2020).

## Results

Following the screening phase, seven guidelines were found, which documented guidelines for chronic pain in the SCI population. Two articles were excluded at the beginning of the critical appraisal phase as one article reviewed guidelines that were published before 2010 (Dworkin et al. 2010), and another was a case report that used the CanPain SCI guidelines (Varghese et al. 2020).

The search results from each database can be found in Appendix 2. Five articles from the literature search and screening process were critically appraised using the AGREE II tool. Table 1 consists of the guidelines reviewed, listed by the author. The table includes the origin of the study, the population and type of pain it was intended for, and the specific guidelines related to the pharmacology and nonpharmacological recommendations.

### Using the AGREE II tool to critically appraise the guidelines

Using the AGREE II appraisal tool, the guidelines by Chetty et al. (2012) and Franz et al. (2019) were classified as high-quality guidelines. In contrast, the guideline by Guy et al. (2016) was classified as an average-quality guideline. Attal et al. (2010) and Schlereth (2020) were classified as low-quality guidelines.

The studies by Franz et al. (2019) and Guy et al. (2016) were the only two studies that aimed their guidelines specifically at the SCI population. However, a distinction between central and peripheral NeuP was not made. The results of using a screening tool to detect a specific pain type can be found in Table 2.

Consistent with the aim of the review to source guidelines for chronic pain (which includes nociceptive pain and NeuP) in the SCI population, only one guideline (Franz et al. 2019) satisfied this aim by recommending treatment for both chronic NeuP and chronic nociceptive pain. This guideline is rated as high quality, scoring above 60% for all the domains on the AGREE II tool. This implies that Franz et al. (2019) correctly addressed the various necessary domains for a guideline. In line with the review’s objectives, the guidelines by Franz et al. (2019) and Guy et al. (2016) included SCI as the specific population. Furthermore, the guidelines by Chetty et al. (2012), Franz et al. (2019) and Schlereth (2020) included recommendations for the use of a screening tool to classify pain before recommending treatment for a specific classification of pain.

Chetty et al. (2012) are also rated as a high-quality article as it addressed all the domains earlier, except the existence of reporting bias due to the authors’ funders. Whether the authors were biassed in their methods and results is unclear; however, the possibility lies in the ‘funding effect’ (Krimsky 2013). This effect is described when bias may exist, as financial conflicts of interest are present. Looking at the study results, the first-line medications, namely pregabalin and gabapentin, are similar to those of a high-quality article by Franz et al. (2019) and, therefore, do not raise the alarm. These drugs are manufactured by one of the funders for the Chetty et al. (2012) guideline. One recommendation, which is not included by any of the other guidelines, is a combination of pregabalin and either an SNRI or amitriptyline as a second-line treatment for chronic pain in the SCI population. In addition, methodological flaws are present in the guideline development, where all the stakeholders met to agree on guidelines; this could have resulted in the introduction of cognitive bias, where stakeholders may have been inclined to agree with others due to group pressure (Thangaratinam & Redman 2005). The anonymity of experts participating in a Delphi study ensures that group pressure or pressure about status or personalities is not introduced (Thangaratinam & Redman 2005).

Guy et al. (2016) were classified as an average-quality guideline as three domains scored above 60% on the AGREE II tool. This guideline omitted to specify the professional designation of each team member involved in the development process (Brouwers et al. 2010). In addition, the guideline is not clear or does not include facilitators and barriers to its implementation, resource restrictions and monitoring or auditing criteria during the use of the guideline (Brouwers et al. 2010). Finally, it is unclear whether the authors were entirely independent of their funders while developing the guideline (Brouwers et al. 2010), as the authors mention that the funder assisted with the guideline development (Guy et al. 2016). This, too, may have introduced reporting bias where the authors may have been inclined to agree with the funder because of their status (Thangaratinam & Redman 2005).

Schlereth (2020) and Attal et al. (2010) were rated as low-quality guidelines as only two or fewer domains scored more than 60%. Two appraisers from the study agreed that the aim of the guideline by Schlereth (2020) is not clearly stated. However, the appraisers disagreed regarding the inclusion and/or omission of health questions and mentioning the actual population for whom the guideline is intended.

The two appraisers agreed that the guidelines were not clear regarding the members of the task team and the users of the guidelines, and it is not apparent whether the guidelines considered the target populations’ views as these were not included or referred to. The two appraisers disagreed regarding including barriers, facilitators, tools and advice for the guideline’s implementation. However, the guideline did not include resource implications or monitoring or auditing criteria for its usage.

The guideline by Attal et al. (2010) scored less than 60% for all domains, apart from the ‘scope and purpose’ domain. The two appraisers disagreed regarding including appropriate stakeholders and target users of the guideline. The two appraisers agreed that the views of the target population were not sought or included. It is clear that there is no procedure described for updating the guideline; the guideline did not undergo external review, and the health benefits versus the risks of the various recommendations are not consistently mentioned (Brouwers et al. 2010). The fundamental recommendations provided by the guideline are not easily identifiable (Brouwers et al. 2010). Lastly, the guideline scored very low (< 40%) for ‘applicability’ and ‘editorial independence’. Barriers and facilitators are not consistently mentioned in the recommendations; the resource implications are unclear; and the tools for implementing the guideline are only provided to a certain degree; that is, first-line and second-line medications are mentioned. However, no additional tools for its implementation, such as titration and maximum dosage, were mentioned. In addition, certain medications lack dosage parameters (Brouwers et al. 2010). Lastly, it is unclear whether the authors were independent in developing the guideline, as the presence and omission of funders is not declared (Brouwers et al. 2010).

## Discussion

This review aimed to gather existing guidelines for chronic pain in the TSCI population and critically appraise these guidelines separately from the scoping review. However, from the initial search, it was evident that no guidelines existed specifically for the TSCI population. Therefore, guidelines were included if they were aimed at the nonspecific SCI population.

The findings show that most guidelines focused on NeuP, and only one guideline (Franz et al. 2019) included nociceptive pain in addition to NeuP. Nociceptive pain of musculoskeletal origin is present following SCI in the form of shoulder, wrist and back pain due to spasms and contractures (Finnerup & Baastrup 2012). A recent systematic review highlighted the burden of chronic musculoskeletal pain, chronic low back pain and chronic back pain in the SCI population (Michailidou et al. 2014). Most of the guidelines (Chetty et al. 2012; Franz et al. 2019; Guy et al. 2016; Schlereth 2020), apart from one (Attal et al. 2010), included nonpharmacological therapy for chronic pain in the SCI population. In addition, the critical appraisal process identified two high-quality articles based on methodological rigour (Chetty et al. 2012; Franz et al. 2019); however, the guideline by Chetty et al. (2012) failed to include treatment for nociceptive pain. The review highlights various recommendations for future research, which will be outlined in the conclusion.

Persons with SCI (nontraumatic) experience central NeuP, whereas some individuals with SCI of a traumatic nature may also experience peripheral NeuP (Hatch et al. 2018). The mechanisms for central NeuP differ from those of peripheral NeuP (Aley & Levine 2002; Finnerup & Jensen 2006; Jensen & Finnerup 2009). Thus, the management principles for peripheral NeuP should differ from those for central NeuP. In addition, certain medications recommended based on the type of NeuP, such as morphine and oxycodone, are recommended both in central and peripheral NeuP conditions. In contrast, botulinum toxin type A-hemagglutinin complex (BoNTA) is only recommended in persons with peripheral NeuP (Szok et al. 2019).

Currently, NeuP is treated symptomatically. However, future treatments should target the underlying pain-generating and pain-maintaining mechanisms (Cavalli et al. 2019). If the mechanisms responsible for pain differ, the authors suggest that the treatment of central NeuP should also vary from that of peripheral NeuP. Despite these variations in mechanisms, the guideline by Chetty et al. (2012) advocates for the use of the same drugs between peripheral and central NeuP due to the lack of available studies using the mechanism-based approach to assessment and treatment. However, the literature suggests that initially identifying a mechanism-based approach to chronic NeuP is the identification of patient symptoms linked to various mechanisms (Bannister et al. 2020).

Rolke et al. (2006) created a quantitative sensory testing protocol (QST), which has gained traction in the last decade as a valuable tool for identifying symptoms in various pain groups and treating these groups of symptoms with specific treatments. However, there are still limitations in applying the QST, such as cost-effectiveness and time required (Cruz-Almeida & Fillingim 2014; Krumova et al. 2012). In the SCI population, the reliability and validity of the QST have been tested in a study by Felix and Widerstrom-Noga (2009), which indicates the support for the use of this tool in the SCI population, despite the study’s small sample size. Future clinical trials should implement the QST protocol on the SCI and/or TSCI population and assess the difference between NeuP types (central versus peripheral) and the pain mechanisms present for the different aetiologies.

In a recent review by Szok et al. (2019), the most effective medication for chronic pain after peripheral nerve injury was tricyclic antidepressants, such as amitriptyline (target descending serotonergic and noradrenergic pathways). The guidelines for chronic pain in the SCI population (Chetty et al. 2012; Franz et al. 2019) propose pregabalin and gabapentin (which act on calcium channels on terminals in the spinal neuronal circuits) as first-line therapy for central NeuP. Chetty et al. (2012) propose amitriptyline as a third option for treatment, whereas Franz et al. (2019) propose amitriptyline as a second-line therapy recommendation. Studies should assess the impact of these medications on peripheral NeuP and central NeuP severity.

In a clinical trial (Rowbotham et al. 2003) assessing opioids of high strength on the impact of chronic pain in persons with central NeuP and peripheral NeuP, a 55% reduction in pain was found in the peripheral NeuP group and a 31% pain reduction in the central NeuP group. However, the number of patients in each group varied greatly, with four in the central NeuP group and 26 in the peripheral NeuP group. Studies assessing the use of opioids are often accompanied by dropout due to the adverse side effects of its usage (Rowbotham et al. 2003). Thus, the recommendation of opioids should be made with caution.

The two guidelines (Chetty et al. 2012; Franz et al. 2019) include considerations and recommendations for multimodal and psychotherapy treatment for managing chronic pain in adults with TSCI and SCI. Chetty et al. (2012) recommend CBT in combination with physiotherapy and pharmacotherapy. On the other hand, Franz et al. (2019) include the fact that psychotherapy, such as imagination, hypnotherapeutic and CBT interventions, in combination with pharmacotherapy, may be considered. The literature demonstrates that cognitive behavioural therapy and mindfulness indicate favourable results in reducing pain, pain-related disability, pain catastrophising, acceptance and coping with chronic pain. However, no comparison is made between various types of pain, such as nociceptive versus central NeuP versus peripheral NeuP (Burke et al. 2019; Hearn & Finlay 2018; Heutink et al. 2014). In addition, depression and a sense of coherence are significantly impacted by a comprehensive programme, which consists of cognitive behavioural therapy and educational sessions, in the SCI population (Budh, Kowalski & Lundeberg 2006). This is a promising field of research, and more investigation should be conducted in the SCI population, specifically among various pain types and etiologies of SCI.

For nociceptive pain of musculoskeletal origin, evidence indicates that physical activities, including stretching and resistance training, have beneficial effects in improving pain, such as low back and shoulder pain (Boldt et al. 2014; Ditor et al. 2003; Geneen et al. 2017; Lewis et al. 2007; Nawoczenski et al. 2006). Physiotherapeutic techniques such as massage and heat improved chronic nociceptive pain in the SCI population (Norrbrink Budh & Lundeberg 2004; Widerström-Noga & Turk 2003). However, these studies do not compare the physiotherapy techniques for various types of pain. Future studies should assess the effects of physiotherapy across multiple types of pain and aetiologies of SCI. One study (Ris et al. 2017) evaluated the variation in nociceptive pain in a population of persons with chronic neck pain of a traumatic nature versus a nontraumatic nature. Outcomes were negatively significantly impacted in the traumatic group compared to the nontraumatic group, precisely the cervical muscle function (reduced strength) and pressure pain threshold. In addition, self-reported function, mental health, quality of life and depression also showed differences between groups, with the traumatic group more adversely affected than the nontraumatic group (Krumova et al. 2012). This finding may be necessary when treating nociceptive pain in the TSCI population versus the SCI population when setting rehabilitation goals and managing mental health. However, additional evidence is required to confirm the difference in mechanisms responsible for traumatic nociceptive pain versus nontraumatic nociceptive pain in the SCI population.

The limitations of the current review include that only articles in English were reviewed, and only open articles were retrieved. In addition, this scoping review did not have a pilot study. However, the authors carefully developed the data extraction tool in line with the purpose and objectives of the study. The first reviewer or first author did not find any difficulty utilising the prefinal extraction tool, and therefore, the authors did not deem it necessary to perform pilot testing.

## Conclusion

One guideline met all the objectives of this scoping review. The guideline by Franz et al. (2019) was explicitly aimed at the SCI population. It referred to a screening tool to identify the type of pain and included pharmacological and nonpharmacological recommendations for different types of pain. This guideline was assessed as a high-quality guideline through the AGREE II tool.

The review highlights the following recommendations for future research: (1) randomised controlled trials should focus on assessing the difference in pain mechanisms between nociceptive pain of a traumatic nature versus nontraumatic in the SCI population; (2) future clinical trials should implement the QST protocol in the SCI and TSCI population and assess the difference between NeuP types (central versus peripheral) as well as the pain mechanisms present for the different etiologies; (3) peripheral NeuP should be differentiated from central NeuP when identifying pain; (4) guidelines should include treatment for chronic nociceptive pain and (5) randomised controlled trials should focus on assessing multimodal and psychotherapy treatment in chronic pain among TSCI and SCI survivors.

By addressing these gaps in research, the future management of chronic pain in the SCI population can be improved.

## Appendix 1

1. Does the citation report guidelines for chronic pain in the SCI population?
  - Yes
  - No, only management
  - No, only treatment
  - Can’t tell
2. Does the citation describe research in English?
  - Yes
  - No
  - Can’t tell
3. Does the citation refer to the spinal cord injury population?
  - Yes
  - No, only spinal cord injury
  - No population described
  - Can’t tell

### Reviewer decision

- If the reviewer’s answer is ‘Yes’ for all questions 1–3, the full text of the article will be retrieved and included for further screening and appraisal.
- If the reviewer’s answer is ‘Can’t tell’ for either or all questions 1–3, the full text of the article will be retrieved and included for further screening and decision-making.
- If the reviewer answers ‘Yes’ to questions 1 and 2 and ‘No, only spinal cord’ to question 3, then the article will be retrieved and included for further screening and appraisal.

## Appendix 2

**TABLE 1-A2:** Database search and results.

Database/platform	Biomed central
Library	University of the Western Cape
Limits	None
Search query	clinical practice guidelines AND chronic pain AND traumatic spinal cord injury OR spinal cord injury
Number of hits	130
**Database/platform**	**Cambridge**
Library	University of the Western Cape
Limits	Only articles Only open access Subjects: Medicine 2010–2022
Search query	clinical practice guidelines AND chronic pain AND traumatic spinal cord injury OR spinal cord injury
Number of hits	2932
**Database/platform**	**Pubmed**
Library	Open access
Limits	2010–2022 Full text
Search query	clinical practice guidelines AND chronic pain AND traumatic spinal cord injury OR spinal cord injury
Number of hits	3
**Database/platform**	**Springerlink**
Library	Open access
Limits	2010–2022 Medicine and public health Subdiscipline: Neurology Articles English only
Search query	clinical practice guidelines AND chronic pain AND traumatic spinal cord injury OR spinal cord injury
Number of hits	76
**Database/platform**	**SAGE Journals Online**
Library	Open access
Limits	2010–2022 Research article Open access content only Health Sciences
Search query	clinical practice guidelines AND chronic pain AND traumatic spinal cord injury OR spinal cord injury
Number of hits	96
**Database/platform**	**Ebscohost, Medline, CINAHL**
Library	University of the Western Cape
Limits	2010–2022 Full text only Adults only Human English
Search query	clinical practice guidelines AND chronic pain AND traumatic spinal cord injury OR spinal cord injury
Number of hits	0
**Database/platform**	**Sabinet Reference**
Library	University of the Western Cape
Limits	2010–2022
Search query	clinical practice guidelines AND chronic pain AND traumatic spinal cord injury OR spinal cord injury
Number of hits	46
**Database/platform**	**ScienceDirect**
Library	Open access
Limits	2010–2022 Nursing and health professionals
Search query	clinical practice guidelines AND chronic pain AND traumatic spinal cord injury OR spinal cord injury
Number of hits	262
**Database/platform**	**Cochrane library**
Library	Open access
Limits	2010–2022
Search query	clinical practice guidelines AND chronic pain AND traumatic spinal cord injury OR spinal cord injury
Number of hits	1
**Database/platform**	**Wiley online library**
Library	Open access
Limits	2010–2022 Open access
Search query	clinical practice guidelines AND chronic pain AND traumatic spinal cord injury OR spinal cord injury
Number of hits	88
Library	University of the Western Cape
Limits	2010–2022 Open access
Search query	clinical practice guidelines AND chronic pain AND traumatic spinal cord injury OR spinal cord injury
Number of hits	3
**Database/platform**	**AHRQ**
Library	Open access
Limits	None
Search query	clinical practice guidelines AND neuropathic pain AND traumatic spinal cord injury OR spinal cord injury
Number of hits	0
**Database/platform**	**AHRQ**
Library	Open access
Limits	None
Search query	clinical practice guidelines AND chronic pain AND traumatic spinal cord injury OR spinal cord injury
Number of hits	0
**Database/platform**	**AHRQ**
Library	Open access
Limits	None
Search query	clinical practice guidelines AND neuropathic pain AND traumatic spinal cord injury OR spinal cord injury
Number of hits	91
**Database/platform**	**AHRQ**
Library	Open access
Limits	None
Search query	clinical practice guidelines AND nociceptive pain AND traumatic spinal cord injury OR spinal cord injury
Number of hits	0
**Database/platform**	**Guideline central**
Library	Open access
Limits	Title
Search query	clinical practice guidelines AND chronic pain AND traumatic spinal cord injury OR spinal cord injury
Number of hits	0
**Database/platform**	**Guideline central**
Library	Open access
Limits	Disease
Search query	Nociceptive pain Neuropathic pain Chronic pain
Number of hits	0
**Database/platform**	**Guideline central**
Library	Open access
Limits	Neurology
Search query	Nociceptive pain Neuropathic pain Chronic pain
Number of hits	0
